# Apical periodontitis associates with cardiovascular diseases: a cross-sectional study from Sweden

**DOI:** 10.1186/s12903-017-0401-6

**Published:** 2017-07-11

**Authors:** Eunice Virtanen, Tapio Nurmi, Per-Östen Söder, Stella Airila-Månsson, Birgitta Söder, Jukka H. Meurman

**Affiliations:** 1 Biomedicum Helsinki, Department of Oral and Maxillofacial Diseases, University of Helsinki and Helsinki University Hospital, P.O.Box 63, Haartmaninkatu 8, FIN-00014 Helsinki, Finland; 2 Department of Dental Medicine, Karolinska Institutet, Huddinge, Sweden; 3 Nordland County Council, Bodö, Norway

**Keywords:** Apical periodontitis, Periodontitis, Cardiovascular diseases, Systemic diseases, Hospital care

## Abstract

**Background:**

Periodontal disease associates with systemic diseases but corresponding links regarding apical periodontitis (AP) are not so clear. Hence our aim was to study association between AP and the prevalence of systemic diseases in a study population from Sweden.

**Methods:**

The subjects were 150 patients from a randomly selected epidemiological sample of 1676 individuals. 120 accepted to participate and their basic and clinical examination data were available for these secondary analyses where dental radiographs were used to record signs for endodontic treatments and AP. Periapical Index and modified Total Dental Index scores were calculated from the x-rays to classify the severity of AP and dental infection burden, respectively. Demographic and hospital record data were collected from the Swedish National Statistics Center. T-test, chi-square and univariate analysis of covariance (ANCOVA) and regressions analyses were used for statistics.

**Results:**

Of the 120 patients 41% had AP and 61% had received endodontic treatments of which 52% were radiographically unsatisfactory. AP patients were older and half of them were smokers. AP and periodontitis often appeared in the same patient (32.5%). From all hospital diagnoses, cardiovascular diseases (CVD) were most common, showing 20.4% prevalence in AP patients. Regression analyses, controlled for age, gender, income, smoking and periodontitis, showed AP to associate with CVD with odds ratio 3.83 (95% confidence interval 1.18–12.40; *p* = 0.025).

**Conclusions:**

The results confirmed our hypothesis by showing that AP statistically associated with cardiovascular diseases. The finding that subjects with AP also often had periodontitis indicates an increased oral inflammatory burden.

## Background

Apical periodontitis (AP) is an inflammatory disorder resulting from failed dynamic encounter between microbial infection of endodontic origin and subsequent host defence response. AP begins as local inflammation in the pulp and the periodontal ligament and grows into larger histopathological lesion characterized by destruction of the periapical tissues [1]. AP represents an infection burden in the populations that varies largely from 17% to 65% in teeth following unsuccessful endodontic treatment [2–6]. The condition is often asymptomatic and its treatment might not always be adequate in eliminating the infection. AP is mainly a radiographic finding. In 2012 a systematic review of cross-sectional studies showed a very high prevalence of periapical radiolucency (1 per patient) and also very high prevalence of root canal treatments (2 per patient), meaning that billions of teeth are kept in the mouth through root canal treatment but with remaining AP and, thus, presenting a potential dental source of infection [7].

Bacteraemia of oral origin occurs at daily activities such as chewing and brushing the teeth; more often so in patients with gingivitis and periodontitis [8]; after dental procedures such as tooth extraction, scaling and root planning, and by non-surgical root canal treatment. Bacteraemia can be detected within 15–30 min in patients who do not have any compromised immune response [9]. Whether bacteraemia is more frequent in AP patients is not known, however.

Periodontitis has been related to a number of different systemic conditions, such as cardiovascular disease (CVD), diabetes, pre-term and low-birth-weight infants [10, 11]. More recently, periodontal disease has been linked to Alzheimer’s disease [12] and also associated with cancer [13]. AP has also been related to CVD [14] and diabetes [15], but data are sparse in this regard. Some studies show the interactions of cytokines resulting from AP lesions with proinflammatory and immunoregulatory mechanisms [16, 17]. A persistent chronic inflammatory condition can influence the cardiovascular system, leading to CVD.

The present study was based on the hypothesis that AP poses a threat to systemic health, similarly to periodontal disease. Consequently, our aim was to study the possible associations between the presence of AP and the prevalence of systemic diseases in general, as registered in the national hospital database from Sweden.

## Methods

### Study population

**Fig. 1:**
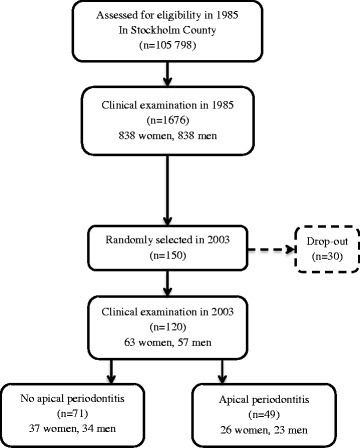
Study population flow-chart

The baseline 1676 subjects had been selected randomly from a larger sample of 3273 subjects, and clinically examined in 1985. In brief, the baseline cohort was selected using the registry file of all inhabitants of the Stockholm metropolitan area. The subjects were born in the 20th of any month between the years 1945–1954. For this cross-sectional study 150 subjects were selected by a computer program from the original epidemiological sample. Of them 120 were willing to attend giving a drop-out of 30 subjects. Those attending were divided into two main groups based on x-ray findings: subjects with and without AP as shown in Fig. 1.

Full mouth x-rays had been taken using Ekta Speed periapical radiographs (Ekta Speed Eastman Kodak, Rochester, NY, USA), an Eggen film-holder and Oralix® or Gendex® Roentgen apparatus (65kVp/7.5 mA) with a cone of rectangular section and a film focus distance of approximately 30 cm [18, 19]. A modified Total Dental Index (TDI) was calculated by recording all signs of infections from the x-rays [20]. The modified TDI scale is from 0 to 10 in recording caries lesions, deep vertical pockets, apical periodontitis, and furcation lesions, respectively; a higher score reflects a greater number of infectious dental diseases and, consequently, a higher total infection burden of the mouth, see Table 1.

**Table 1:** Modified Total Dental Index [20]

Type of disease	Score
Caries	
No caries	0
1–3 carious lesions	1
4–7 carious lesions	2
≥ 8 carious lesions or infected roots or no teeth	3
Periodontitis	
None	0
1–3 deep vertical pockets	1
4–7 deep vertical pockets	2
≥ 8 deep vertical pockets	3
Apical periodontitis	
None	0
1 tooth	1
2 teeth	2
≥ 3 teeth	3
Furcation lesions	
Absent	0
Present	1

**Fig. 2:**
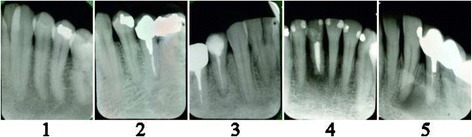
Grading of the Periapical Index: 1 = normal, 2 = bone structural changes, 3 = bone structural changes with signs of mineral loss, 4 = radiolucency, and 5 = radiolucency with features of exacerbation

In the teeth with AP, the Periapical Index (PAI) was calculated to classify the extent and severity of the apical lesions with the following scale: 1 = normal, 2 = bone structural changes, 3 = bone structural changes with signs of mineral loss, 4 = radiolucency, and 5 = radiolucency with features of exacerbation [21]. Examples are shown in Fig. 2. PAI score ≥ 2 indicates disease. Number of root canal treatments, satisfactory and unsatisfactory, respectively, were also registered. Root canal treatment was considered satisfactory when all roots and all root canals of a tooth were filled up to 0–2 mm from the apex. The x-ray analyses were made by two of the authors (EV and TN) using a magnifying viewer (X-Produkter®, Malmö, Sweden) and a standard view box with constant light intensity. Pre-study calibration was not thought necessary. Eventual disagreements were mutually settled. At the time of x-ray examinations no other information about the patients was available, so the examiners were blinded in this regard.

From the earlier conducted clinical examination data made by one of the authors (SA-M), the following parameters were used in the present investigation: number of teeth and number of missing teeth (excluding third molars); gingival inflammation around every tooth (Gingival Index [GI]) [22] and Bleeding on Probing [BOP]); oral hygiene status (Plaque Index [PLI]) [23], and Calculus Index (CI) [24]. Periodontal pocket depth (PD) had been measured with a periodontal probe to the nearest highest point in all six representative surfaces of each remaining tooth, and the clinical attachment loss (CAL) had also been registered. To define periodontal health status, patients with 1 or more deep pockets (≥5 mm) and local bleeding (recorded with BOP index) were considered periodontitis patients (“Perio”), while the others were periodontitis free (“No perio”).

Basic characteristics of the subjects were available. These included age, education, income per year, social status, working status, smoking habits, and frequency of dental visits of each subject. The socioeconomic and hospital data were obtained from the National Statistics Centre, Örebro, Sweden. The socioeconomic data is from 1985 and the hospital data is from 2003. For recording the prevalence of systemic diseases from the database, World Health Organization International Classification of Diseases (ICD-9-10) was used. All patients selected were included in the study and none had taken antibiotics in the previous 6 months.

### Statistical analyses

The statistical analyses were made using the IBM SPSS Statistics 22 program. Independent samples t-test for equality of means was performed to compare the two groups with and without AP, taken into account demographic variables. Two-tailed significance was set at 0.05. Analysis of covariance (ANCOVA) tests were conducted to adjust for age, gender and smoking in the comparisons of the other variables. To verify how apical periodontitis correlate with periodontitis a regression analyses model was designed, with end point apical periodontitis and possible explanatory factors from the socioeconomic data (age, gender, income), smoking habits and clinical indexes related with periodontitis (BOP, GI, PLI, CI, PD and CAL). The number of missing teeth was also inserted in the model. To study the number of AP lesions and their severity, chi-square test was used. Because group comparisons showed that AP patients had more often been hospitalized because of CVD, a regression model was constructed to evaluate the associations between the independent variables age, gender, income, smoking habits, periodontitis, AP and number of missing teeth, and the dependent variable “cardiovascular diseases”. The independent variables were selected for being factors that can influence the cardiovascular condition of the patients, such as age, gender, income and smoking habits. From the oral cavity, periodontitis scores and apical periodontitis scores represent the present infection burden in the mouth while the number of missing teeth represents past infection burden of the mouth. Backward stepwise likelihood ratio method for analyses was used.

## Results

Of the 120 patients 40.8% had AP as recorded from the x-rays. The mean incidence was 0.74 with a standard deviation (SD) of 1.03. Of the patients 17.5% had one apical lesion, 13.3% two apical lesions and 10% three or more apical lesions. Endodontic treatment had been given to 60.8% of the patients; mean prevalence was 1.93 (SD 2.27). Of these 38.3% were assessed as satisfactory while 51.7% were recorded as unsatisfactory. In 10% it was not possible to evaluate the quality of endodontic treatments because of poor quality x-rays.

Table 2 gives the comparison between the patients with and without AP. Those with AP were older and significantly more often smokers. Women showed more often AP than men. Patients with AP had significantly lower income while no other difference was observed in the socio-economic variables between the groups.

**Table 2:** Demographic, clinical and X-ray data with respect to apical periodontitis

	No apical periodontitis (*n* = 71)	Apical periodontitis (*n* = 49)	
	Number, mean ± SD	Number, mean ± SD	p*
Age (years)	**51.44 ± 2.92**	**52.98 ± 2.73**	**0.001**
Gender (women/men)	**37/34**	**26/23**	**0.028**
Education (higher/compulsory)	59/12	42/7	NS
Income (Swedich crowns ×1000)	**1955 ± 860**	**1734 ± 654**	**< 0.001**
Social status (high/low)	58/13	40/9	NS
Working status (working/not working)	65/6	48/1	NS
Smoking (yes/no)	**17/54**	**25/24**	**0.009**
Dental visits (years interval)	1.37 ± 0.76	1.53 ± 0.79	NS
Hospital visits (yes/no)	23/48	20/29	NS
Periodontal status (Perio/No perio)	**44/27**	**39/10**	**0.003**
Gingival Index	**0.76 ± 0.96**	**1.25 ± 1.04**	**< 0.001**
Plaque Index	**0.31 ± 0.31**	**0.47 ± 0.52**	**< 0.001**
Calculus Index	**0.83 ± 0.10**	**0.19 ± 0.46**	**0.033**
Bleeding on probing	0.26 ± 0.22	0.34 ± 0.24	NS
Clinical attachment loss (mm)	**2.73 ± 0.89**	**3.40 ± 1.75**	**< 0.001**
Probing depth (mm)	**2.38 ± 0.61**	**2.82 ± 0.91**	**< 0.001**
Total Dental Index (TDI)	**1.03 ± 1.42**	**3.57 ± 1.73**	**< 0.001**
Caries (TDI)	0.23 ± 0.54	0.35 ± 0.63	NS
Perio (TDI)	**0.61 ± 1.04**	**0.90 ± 1.10**	**0.015**
Furcation (TDI)	**0.20 ± 0.40**	**0.51 ± 0.51**	**< 0.001**
Root treatments	**0.99 ± 1.58**	**3.31 ± 2.43**	**< 0.001**
Satisfactory root treatments	**0.38 ± 0.74**	**0.98 ± 1.05**	**< 0.001**
No satisfactory root treatments	**0.58 ± 1.16**	**2.33 ± 1.87**	**< 0.001**
No. of missing teeth	**1.59 ± 1.83**	**3.45 ± 4.55**	**0.001**
No. of missing molars	**0.69 ± 1.13**	**1.71 ± 1.90**	**< 0.001**

Data is expressed as mean ± SD

*Significance adjusted with UNIANOVA for age, gender and smoking, NS *p* > 0.05

Significant results are given in bold (*p* ≤ 0.05)

In general, patients with AP had higher periodontal index scores than those without AP. The results are also given in Table 2. Furthermore, x-ray data showed significantly higher TDI scores for periodontitis markers and higher number of furcation lesions among the AP patients but no difference could be seen between the groups in the number of caries lesions. The number of root canal treatments and particularly unsatisfactory endodontic treatments were higher in the AP patients. In patients without AP endodontic treatments had also been given, and a number of those were recorded as unsatisfactory in quality, but still there were no x-ray signs of AP. Patients with AP also had more teeth missing, especially missing molars when compared with those without AP lesions.

In the regression analysis for studying the association between AP and periodontitis, age, PD, CAL and missing teeth were the last variables to remain and were the significant explanatory factors for having apical periodontitis. The results are given in Table 3.

**Table 3:** Results from logistic regression analyses with the dependent variable “apical periodontitis” and a number of possible explaining variables (age, gender, income, smoking habits, BOP, GI, PLI, CI, PD, CAL and missing teeth)

Dependent variable	Explanatory variable	Beta	Chi-Square	*p*-value	OR (95% CI)
	Age	0.21	7.89	0.005	1.24 (1.07–1.44)
Apical Periodontitis	PD	1.69	6.52	0.011	5.43 (1.48–19.87)
	CAL	0.95	4.27	0.039	2.60 (1.05–6.41)
	Missing teeth	0.27	6.34	0.012	1.31 (1.06–1.61)

Cox & Snell R^2^ = 0.18; Nagelkerke R^2^ = 0.24

Table 4 gives the hospital care data of the registered systemic diseases. Of these CVD diagnosis was the most common. Of the AP patients 20.4% had been in hospital for treatment of CVD. Next in prevalence were neoplasms, infectious and parasitic diseases. The individual cardiovascular diagnoses were manifold; hypertension being the most common diagnosis.

**Table 4:** Systemic diseases registered in the subjects during hospital care without and with apical periodontitis

Group of the diseases in ICD-9 and ICD-10	No apical periodontitis (n)	%	Apical periodontitis (n)	%
Cardiovascular diseases	6	8,5	10	20,4
Benign neoplasms	5	7,0	3	6,1
Malignant neoplasms	5	7,0	2	4,1
Infectious and parasitic diseases	5	7,0	1	2,0
Endocrine, nutritional and metabolic diseases	1	1,4	2	4,1
Diseases of the nervous system	1	1,4	2	4,1
Diseases of the musculoskeletal system and connective tissue	1	1,4	1	2,0
Diseases of the blood and blood-forming organs	1	1,4	0	0
Diseases of the genitourinary system	1	1,4	0	0
Mental disorders	1	1,4	0	0
Immunity disorders	1	1,4	0	0
Symptoms, signs and abnormal clinical and laboratory findings, not elsewhere classified	1	1,4	0	0

In the regression analysis with the dependent variable “cardiovascular diseases”, and age, gender, income, smoking habits, periodontitis, AP, and number of missing teeth as independent variables, a significant association was found between AP and CVD, with odds ratio 3.90 (95% confidence interval 1.20–12.65; *p* = 0.023). Age was the other independent factor in the last step but it was not significant anymore (*p* = 0.061). The results are given in Table 5.

**Table 5:** Results from logistic regression analyses with the dependent variable “cardiovascular diseases” and a number of explaining variables (age, gender, income, smoking habits, periodontitis, apical periodontitis, and missing teeth)

Dependent variable	Explanatory variable	Beta	Chi-Square	*p*-value	OR (95% CI)
Cardiovasvular diseases	Apical periodontitis	1.34	5.02	0.025	3.83 (1.18–12.40)
	Age	0.19	3.52	0.061	1.21 (0.99–1.48)

Cox & Snell R^2^ = 0.06; Nagelkerke R^2^ = 0.11

**Fig. 3:**
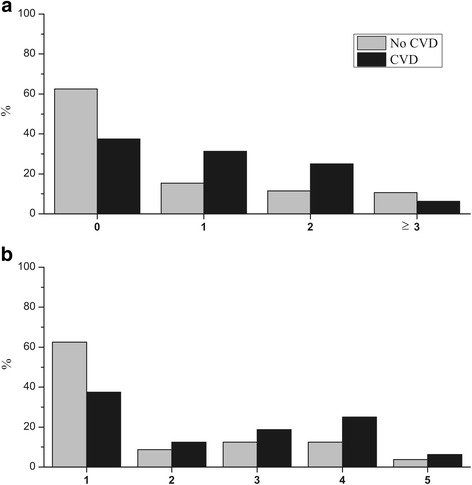
**a** Number of AP lesions, based on TDI scores, and **b**, severity of AP lesions, based on PAI scores, in patients without and with cardiovascular diseases (CVD). No statistically significant difference between the groups was observed, but there was a tendency showing that CVD patients have more lesions and more severe lesions compared to no CVD patients

Figure 3 shows a tendency that AP lesions were indeed more prevalent in patients with than without CVD and that AP lesions also were more severe among these patients, as assessed using the PAI scoring. However, here the difference between the groups was not statistically significant.

## Discussion

We investigated the prevalence of AP in a Swedish study population to find out if or not AP associates with the prevalence of any systemic disease as recorded in the cumulated national hospital database. The study hypothesis was based on the oral infection – systemic disease paradigm.

The prevalence of AP was 41% in our subjects which is within the range reported in other studies from Sweden [4, 25, 26]. This still is a high number of lesions anticipated to pose a threat to health. The prevalence of root canal treatments was high (61%) but the biggest problem was their poor quality (52%). An earlier study from Sweden shows that there was no improvement of the periapical status of root filled teeth, even though the x-ray quality of the root canal fillings was reported to have improved from year 1973 to 2003 [4]. In the present study, our evaluation was only based on the radiographic analysis. There were no data available about treatment methods and materials. Neither did we know when the endodontic treatments had been given to the patients. These are among the limitations of the present study. The strengths, however, were the representative original study sample and the unique national database used in this study. Each individual hospitalization and its reason are recorded in the register in Sweden.

AP patients had multiple dental problems as indicated by the high TDI score. Furthermore, our results showed a clear connection between AP and periodontitis, as observed in high scores in most of the periodontal indexes recorded from the AP patients. The regression model showed that PD and CAL indeed were significant explanatory factors for having apical periodontitis. The same was found in the x-ray analyses where signs of periodontitis and furcation lesions in particular were significantly more frequent in AP patients. Recently, a significant association between periodontitis and AP was reported in a study also conducted in Sweden [27]. Hence, these two pathological entities are more similar to each other than maybe earlier thought. They both are polymicrobial infections sharing similar microbiota, they often are chronic in nature, and can cause up-regulation of cytokines and other inflammatory mediators which, in turn, may have systemic consequences [15, 28]. Indeed, our results showed that CVDs were more common in patients with AP and, in particular, among patients who also had concomitant periodontitis. In the AP patients the significantly higher values of PLI and GI scores indicate poor oral hygiene with consequently higher values of PD and CAL. The results might imply that chronic inflammation had been persistent for a long time in the periodontal ligament causing a higher oral infection burden to the patients. We may speculate that also the higher number of missing teeth here observed might reflect both AP and periodontitis in the patients’ earlier life since teeth are commonly extracted mainly because of infection. AP patients in our study indeed had significantly more missing teeth than patients without AP, especially molars were more often missing.

The prevalence of other systemic diseases than CVDs among the patients was more scattered. No statistically significant differences could be observed between the groups in this regard and in general the prevalence figures were low. At the time of the present examination of the subjects they were at most 58 years old. In that age malignant diseases, for example, are still rare. On the other hand our present study sample was small.

Even though the age range of our patients was only 10 years by definition at the inclusion, the significant difference between the groups showed that age, also here, plays an important role in AP. In addition, those with AP were more often smokers. This can be expected to affect periapical tissue metabolism the same way as is known for periodontitis [29]. However, in earlier studies there is a discrepancy whether or not smoking affects AP [30, 31]. More studies are thus called for in this area.

In our study population AP was more often recorded from women which was contrary to a study conducted in Finland, where the prevalence of apical periodontitis was higher in men [32]. Of the socio-economic variables the lower income observed among the AP patients may explain why their chronic dental diseases had not been properly treated. Costs of the treatment to the patient might have been an obstacle here.

In recent years several studies on AP and systemic health have been published suggesting an association between endodontic variables and systemic diseases and, consequently; the need for “endodontic medicine” to address all those issues has been discussed [15]. Poor oral health in general and the presence of root canal treatments and endodontic infections specifically associate statistically with CVD [14, 33] and, specifically with coronary artery disease [34]. The importance of eliminating oral infectious foci for controlling glycaemia in patients with diabetes mellitus has also been vastly discussed [35]. Furthermore, in pregnant women AP seems to associate with shorter pregnancy duration and with intrauterine growth restriction [36]. The impact of AP systemically can be by the alteration of serum levels of different cytokines and nitric oxide, as has been shown in rat models [37]. Hence endodontic infections need indeed be considered simultaneously with other dental infections under the paradigm of oral infections and general health.

The small number of patients was the main weakness in this study. It also might explain why some differences between groups, such as shown in Fig. 3, were statistically not significant. We used PAI is the scoring in the classification of the severity of AP lesions based on apical radiographs. In terms of biological effect, however, other methods such as 3D radiographs or taking histological samples, might be better regarding assessment of the severity of the lesions, but these were not available for the present examination.

## Conclusions

The results from our study showed that AP statistically associates with prevalence of CVD. No causal relationship can be discussed in this connection, however, where our results were based on secondary cross-sectional analysis. The results nevertheless confirmed our study hypothesis and emphasizes the need for eliminating local infections that may increase the systemic infection burden.

## Abbreviations

AP: Apical periodontitis
BOP: Bleeding on probing
CAL: Clinical attachment loss
CI: Calculus index
CVD: Cardiovascular diseases
GI: Gingival index
PD: Probing depth
PLI: Plaque index
